# Frailty Screening in the Emergency Department: Comparing the Variable Indicative of Placement Risk, Clinical Frailty Scale and PRISMA-7

**DOI:** 10.3390/ijerph20010290

**Published:** 2022-12-24

**Authors:** Rónán O’Caoimh, Jane McGauran, Mark R. O’Donovan, Ciara Gillman, Anne O’Hea, Mary Hayes, Kieran O’Connor, Elizabeth Moloney, Megan Alcock

**Affiliations:** 1 Mercy University Hospital, Grenville Place, T12 WE28 Cork, Ireland; 2 Clinical Research Facility Cork, University College Cork, Mercy University Hospital, T12 WE28 Cork, Ireland

**Keywords:** emergency department, frailty, screening, clinical frailty scale, diagnostic accuracy, variable indicative of placement risk, PRISMA-7

## Abstract

Prompt recognition of frailty in the emergency department (ED) is important to identify patients at higher risk of adverse outcomes. Despite this, few studies examine the diagnostic accuracy of screening instruments for frailty, instead focusing on predictive validity. We compared three commonly used, short frailty screens to an independent comprehensive geriatric assessment (CGA) in an urban University Hospital ED. Consecutive attendees aged ≥70 years were screened by trained raters, blind to the CGA, with the Variable Indicative of Placement risk (VIP), 3 and 4-item versions, Clinical Frailty Scale (CFS) and PRISMA-7. Accuracy was measured from the area under the ROC curve (AUROC). In total, 197 patients were included, median age 79 (±10); 46% were female. Half (49%) were confirmed as frail after CGA. All instruments differentiated frail from non-frail states, although the CFS (AUROC: 0.91) and PRISMA-7 (AUROC: 0.90) had higher accuracy compared to the VIP-4 (AUROC: 0.84) and VIP-3 (AUROC: 0.84). The CFS was significantly more accurate than the VIP-3 (*p* = 0.026) or VIP-4 (*p* = 0.047). There was no significant difference between the CFS and PRISMA-7 (*p* = 0.90). The CFS and PRISMA-7 were more accurate and should be considered in preference to the VIP (3 or 4-item versions) to identify frailty in EDs.

## 1. Introduction

Population ageing is associated with increased mortality [1]. Such ageing is also associated with a high prevalence [2] and incidence [3] of frailty, an age-associated vulnerability to adverse outcomes [4]. Currently, older patients account for approximately one-fifth (22%) of hospital emergency department (ED) visits, and ED resource use intensity increases with age [5]. Older patients often have multiple comorbidities, a greater proportion of complex presentations and are more frequently admitted to hospital [6]. Demographic projections suggest that there will be an increase in the number of older patients presenting to ED [7]. This is expected to put further pressure on already strained healthcare systems requiring increased hospital resources in terms of staffing, resource use and bed capacity. It is therefore important to put processes in place to improve the management of older persons presenting to the ED.

Determination of frailty status is useful to inform clinical decisions, as frailty scores can predict multiple adverse health-related outcomes following hospitalisation [8]. This in turn helps highlight patients that may benefit from admission to dedicated geriatric medicine units [9] and from targeted integrated interventions [10]. Multiple short frailty screening instruments are available for use in ED [11]. Despite this, frailty screening is only performed with approximately half of eligible older patients in ED [12]. The reasons for this are complex. Some healthcare providers lack education on this topic and rely on clinical judgement [13]. Where available, training on how to administer and score frailty screens is often sub-optimal [14]. The time taken to complete screening is also reported as a barrier [13], relating to the well-established trade-off between administration time and accuracy, inherent to the use of any screening instrument [15]. To date, no single instrument is recommended by experts to screen for frailty in the ED [16] and few studies have examined the diagnostic accuracy of screening tools for independently determined frailty. Instead, they focus on predictive validity for adverse health outcomes such as mortality [17]. This limits the generalisability and comparability of these studies [18,19]. Further, only half of studies compare established screens with each other with most examining predictive validity of a single instrument recorded in ED [18].

Given these points, we compared the ability of three of the most commonly used, short frailty screening tools found as part of the development of a systematic review of such instruments [17]: the Clinical Frailty Scale (CFS) [20], the Variable Indicative of Placement risk (VIP) [21] and the Programme on Research for Integrating Services for the Maintenance of Autonomy 7 item questionnaire (PRISMA-7) [22], to correctly identify independently verified frailty in the ED using the most widely-recognised “gold standard” comprehensive geriatric assessment (CGA), conducted by a consultant geriatrician-led inter-disciplinary team. The ability of each screening tool to predict negative health-related outcomes including admission, readmission, length of hospital stay (LOS) and deaths were also assessed.

## 2. Materials and Methods

### 2.1. Patients

Older adults aged ≥70 years, presenting consecutively to a single, urban (inner city) university hospital ED in the South of Ireland over a four-week period in the autumn of 2019, were screened for frailty. Screening was performed between 9 am and 5 pm, Monday to Friday inclusive. Patients were included if they were community-dwelling and medically stable based on a Manchester Triage System (MTS) score of greater than one on admission. The MTS uses five levels to prioritise patients at ED triage from level one (immediate) to five (non-urgent) [23]. Those deemed to be unstable, i.e., requiring management in the ED resuscitation, cardiac care or intensive care units with MTS score of one or at end-of-life, were excluded. Patients in residential care, where frailty prevalence is high [24] were similarly excluded. Patients who were scheduled to return to ED for a planned review were excluded. Finally, patients who were agitated such as they were unable to be assessed, were lost to follow-up and those who declined were also excluded. Ethics approval was obtained in advance from the local ethics committee (Cork Teaching Hospitals reference ECM 3 (uuu) 25/07/19). Patients provided written, informed consent. Where this was deemed not possible by the principle investigator, verbal assent was sought and the family informed, where appropriate.

### 2.2. Instruments

The VIP is a brief, frailty-orientated risk-prediction screen, originally validated in Belgium among ‘non-geriatric’ patients [21]. Quick to administer, it is a three and/or four question tool that can be scored by someone without training in geriatric medicine [21]. It correlates positively with LOS and can identify patients who do not need geriatric assessment [21]. While used in many countries as a short frailty screen in ED, it has yet to be formally validated in this setting [19]. The VIP comprises three (VIP-3) or four (VIP-4) questions. The questionnaire includes a social question (whether the patient is living alone), physical item (autonomy in washing and dressing), a cognitive item (using the telephone) and a general measure of independence (moving around the neighbourhood independently) [25]. A score of ≥2 on the VIP-3 or ≥3 on the VIP-4 identifies patients at increased risk for discharge problems, loss of functional independence and increased LOS [21,22,25]. The CFS classifies patients according to their level of frailty using clinical judgement, taking into account their recent (two-week) baseline function [20]. It is validated in ED to measure frailty [26] and predict inpatient mortality, prolonged LOS and admission to geriatric wards [9]. The CFS combines images and written clinical descriptions and is scored from one (very fit) to nine (terminally ill). A score of four defines living with ‘very mildly frail’ (pre-frail), while a cut-off of five or more is taken as ‘living with frailty’ [20]. The PRISMA-7 is a seven-point questionnaire requiring yes or no answers that includes demographic information (age and sex), physical ability, if there are medical problems that limit the patient and if there is dependence on others. It is scored between 0–7 points, with higher scores indicating higher severity of frailty. A cut-off of ≥3 suggests the need for further assessment [22].

### 2.3. Data Collection

In advance of to the study, ED nurses received standardised information on frailty (lectures and handouts) and were trained to score the study instruments. Education was provided to staff over a week, in-between their shifts. Screening was completed after the standard ED triage. The ED nurse scored patients with the screens in random order. Where patients were deemed unable to comply due to sensory or cognitive impairment, caregivers, family, or those attending with patients, where available and with permission of the participant, were invited to assist. Test scores of all three instruments were then concealed. A dedicated multi-disciplinary frailty team (MDT) including a geriatric consultant, a physiotherapist, an occupational therapist, two geriatric registrars and a geriatric advanced nurse practitioner completed a CGA (described below) on all those screened, blind to the screening test scores. Similarly, those conducting the screening were unaware of the result of the CGA. The consultant adjudicated all cases to ensure quality control. Hospital admission, LOS if admitted, 30 and 90-day readmissions (to either the regional model 3 or 4 hospital) and three-month mortality (including inpatient death) data were obtained from hospital electronic records.

### 2.4. Measures

Frailty status (frail versus non-frail) was assessed following an independent CGA, conducted by the MDT, based on a battery of assessments. This was the primary outcome and a selection of different assessments including other frailty measures, quality of life and cognitive and nutritional measures were obtained. These were conducted blind to (i.e., independent of) the results of the screens. Medical records and medication lists were also reviewed. Where available, a collateral history was obtained and family members asked to complete a six item Caregiver Burden Score (CBS) applying a cut-off of ≥15/30 for burden [27]. This battery included two validated frailty measures: the FRAIL Scale, measuring physical frailty [28], and the Groningen Frailty Indicator (GFI), which includes multi-domain elements of frailty [29]. The FRAIL scale ranges from 0–5 (from non-frail to increasing frailty), with scores of 1 or 2 suggesting pre-frail and ≥3 as frail. The GFI asks yes/no questions incorporating cognition, physical function, social and psychological factors relating to frailty. Moderate to severe frailty is defined by a cut-off score of ≥4 from a total of 15 points. These were used in the subsequent analysis to examine the accuracy of the short frailty screens for other types of frailty, e.g., physical frailty based on the FRAIL scale cut-off of ≥3. The battery also included the Mini-Nutritional Assessment-short form (MNA-SF), taking a cut-off score of ≤11 for risk of malnutrition [30]. This was supported by the patient’s body mass index (BMI). The Euroqol EQ-5D visual analogue scale scored from 0 to 100 (i.e., from worst to best imaginable health state today) [31] and the general self-rated health (GSRH) item on the SF-36 instrument [32] were used to measure quality of life (QOL). The 4AT and AD8 were used to identify delirium and cognitive impairment, respectively. 4AT scores of 1–3 are indicative of possible cognitive impairment, while scores > 4 suggest delirium +/− cognitive impairment [33]. A cut-off of ≥2/8 on the AD8, supported a diagnosis of cognitive impairment [34].

### 2.5. Statistical Analysis

A sample size calculation was performed a priori, based upon previous research conducted by this group [26], suggesting that frailty screening instruments have an expected sensitivity and specificity of approximately 80% [26], taking an expected prevalence of frailty among those aged ≥70 years attending ED of 50% [26], with 95% confidence intervals (CI) at a precision (margin of error) of ±0.10, yielded a recommended sample size of participants 123, which allowing for a 10% drop-out rate (decline to consent), gave a target sample size of 137 [35,36]. Data were analysed with SPSS V26 (Chicago, IL, USA) and Microsoft Excel Version 2005. The Shapiro–Wilk test was used to test normality and found that all main variables were non-parametric at a cut-off of *p* < 0.05. The Mann–Whitney U test was used to test the statistical significance of the association between frailty status and non-parametric variables. Statistical significance was tested for binary variables using the Pearson’s chi-square test. Spearman’s correlation coefficient (r_s_) was used to test the agreement between frailty scales (number of criteria); and Cohen’s kappa (κ) for the agreement between the frailty cut-offs. The sensitivity, specificity, positive predictive value (PPV) and negative predictive value (NPV) of each screen were calculated at different cut-offs. The accuracy of frailty scores was assessed from the area under the curve (AUC) of receiver operating characteristics (ROC) curves, compared with the DeLong method [37]. The optimal cut-off was calculated using Youden’s Index (J = Sensitivity + Specificity − 1).

## 3. Results

During the time period of this study, from the 14 August 2019 until the 12 September 2019, there were 2867 patient presentations to the ED. Of these patients, 492 (17%) were aged ≥70 years and 225 (46%) of these were screened. From these 197 (40% of those ≥70) were included in this analysis. Several patients (*n* = 28) were excluded for reasons including being too unwell (*n* = 4), residing in nursing homes (*n* = 10), patient refusal (*n* = 1), and unable to be assessed (*n* = 6), previously screened on another visit (*n* = 2), and those that were lost to follow-up (*n* = 5).

The characteristics of these 197 patients are provided in Table 1 by frailty status. The median age for the sample was 79 years (IQR: 73–83), and 90 (46%) were female. While there were more females in the frail group this difference was not statistically significant (49% vs. 41%, *p* = 0.257). As seen in Table 1, those categorized as frail (50%) according to the CGA (*n* = 98/197) were significantly older (median 80 vs. 75 years, *p* < 0.001), had lower BMI (median 24 vs. 26, *p* = 0.009) and more malnutrition (MNA-SF median 11 vs. 13, *p* < 0.001). They were also more likely to screen positive for dementia on the AD8 (*p* < 0.001), have possible delirium on the 4AT (*p* < 0.001) and to score higher for caregiver burden (CBS median 15 vs. 0, *p* = 0.001). Patients identified as frail reported overall worse health status (EQ-5D median 50 vs. 70, *p* < 0.001) and were less likely to describe their health as very good or excellent (6% vs. 35%, *p* < 0.001).

In total, 66% of the sample were admitted and these had a median LOS of seven days. At three months 14 (7%) of patients had died. By 90 days 55 (28%) had either re-presented to ED or were re-admitted to hospital. Those categorised as frail were more likely to be admitted (hospital admission rate of 82% vs. 49%, *p* < 0.001), have increased LOS if admitted (median 9 vs. 4 days, *p* = 0.003) and had reduced three-month survival (12% vs. 2% mortality, *p* < 0.005), although there were no statistically significant differences in re-attendance rates, irrespective of how this was defined.

All frailty screening instruments had higher median scores for participants that were categorised as frail according to CGA than as non-frail (Table 1). There was strong correlation between the CFS and PRISMA-7 scores (r_s_ = 0.70), but only moderate correlation between CFS and VIP (r_s_ = 0.56–0.58) and PRISMA-7 and VIP (r_s_ = 0.48–0.51). Agreement between each frailty screen (using accepted cut-offs) and patient frailty status was strongest for the CFS (κ = 0.64) and PRISMA-7 (κ = 0.62) followed by the VIP-3 (κ = 0.56) and VIP-4 (κ = 0.29), see Table 2. Agreement and correlation with the frailty measures used as part of the CGA (i.e., the FRAIL scale and GFI) are also included.

**Figure 1:**
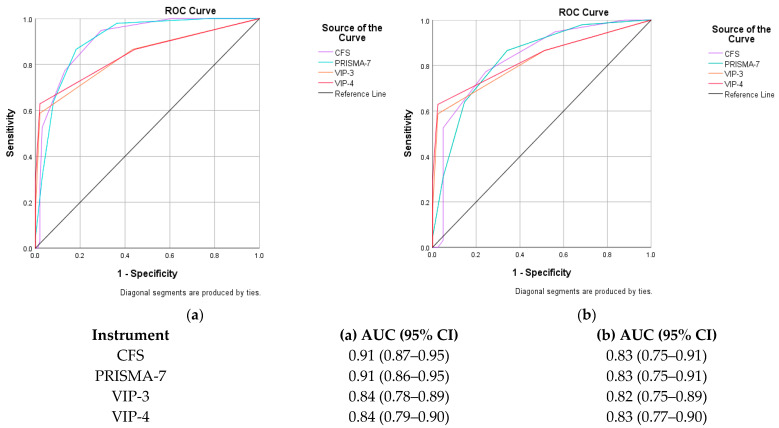
Receiver operating characteristic curves with area under the curve scores showing the accuracy (area under the curve) of the Clinical Frailty Scale (CFS), FRAIL scale, Groningen Frailty Index (GFI), Programme of Research to Integrate Services for the Maintenance of Autonomy 7 (PRISMA-7) tool, three-item Variable Indicative of Placement risk (VIP-3) and the and the four-item Variable Indicative of Placement risk (VIP-4) in differentiating (**a**) frail from non-frail and (**b**) frail from pre-frail older adults in the Emergency Department (classification based on the comprehensive geriatric assessment).

ROC curves examining the diagnostic accuracy for separating frailty from non-frailty (robust and pre-frail) are presented in Figure 1a. Analysis found that the CFS (AUC: 0.91, 95% CI: 0.87–0.95) and the PRISMA-7 (AUC: 0.91, 95% CI: 0.86–0.95) were better at distinguishing frail participants from those that were non-frail compared to the VIP-3 (AUC: 0.84, 95% CI: 0.78–0.89) or VIP-4 (AUC: 0.84, 95% CI: 0.79–0.90). The CFS was statistically significantly more accurate than the VIP-3 (*p* = 0.026) and VIP-4 (*p* = 0.047) in identifying frailty. There was no significant difference between the CFS and PRISMA-7 (*p* = 0.90). All screens performed similarly (AUC between 0.82–0.83) in their ability to distinguish patients that were frail from those who were pre-frail (Figure 1b). Table 3 provides the optimal cut-off scores and psychometric properties for each screen for differentiating frailty states according to the independent CGA. The CFS had an optimal cut-off ≥ 4 for frailty based on Youden’s Index, providing a sensitivity of 95% (CI: 0.88–0.98) and specificity of 70% (CI: 0.60–0.79). At the established cut-off of ≥4 it was less sensitive (77%) but highly specific (87%). The VIP (3 or 4-item) had generally low sensitivity but had high specificity.

**Figure 2:**
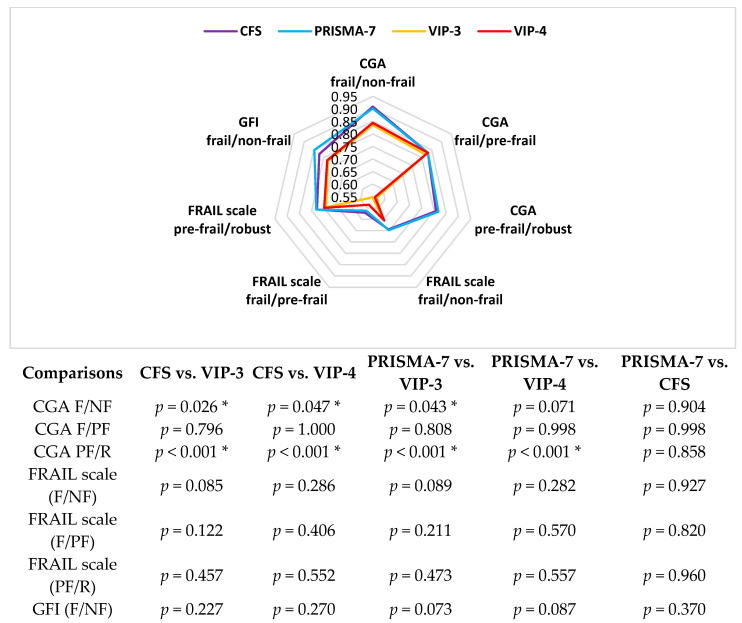
Comparison of area under the curve scores between the Clinical Frailty Scale (CFS), the Programme of Research to Integrate Services for the Maintenance of Autonomy 7 (PRISMA-7) tool, the three-item Variable Indicative of Placement risk (VIP-3) and the four-item Variable Indicative of Placement risk (VIP-4) for differentiating participants who were frail from non-frail (F/NF), frail from pre-frail (F/PF), and pre-frail from robust (PF/R), with *p*-values illustrating the statistical significance of differences between the AUC of each tool. * Significance tests (*p* < 0.05).

A visual summary of the diagnostic accuracy of each frailty screen is presented in Figure 2 with hypotheses testing for the difference in the AUC between each. Comparing screens, all had greater accuracy for frailty as measured using the CGA than for physical frailty as measured using the FRAIL scale (poor to fair). These data are presented in Table 4 alongside the predictive accuracy of the screens for adverse outcomes. The screens all had lower accuracy for separating pre-frail from non-frail and again this was lowest for the VIP (Table 4). Examining adverse health outcomes, while all were significant predictors of whether the patient was admitted from ED (compared with chance alone), the accuracy was at best fair and was highest for the CFS (AUC: 0.78, *p* < 0.001) and lowest for the VIP-3 (AUC = 0.59, *p* = 0.032). The CFS was statistically significantly more accurate in predicting whether patients were admitted than either the VIP-3 or VIP-4 (*p* < 0.001) or PRISMA-7 (*p* < 0.001); there was no difference between the PRISMA-7 and the VIP. Based on the median LOS (7 days), all screens were poor predictors of prolonged LOS with no statistically significant difference between them. Similarly, they were all poor predictors of risk of re-admission. Finally, only the CFS (AUC = 0.71, *p* = 0.008) and PRISMA-7 (AUC = 0.71, *p* = 0.009) had fair, albeit statistically similar accuracy in predicting death at 3 months (*p* = 0.911). Neither were significantly more accurate than the VIP.

## 4. Discussion

This study presents a psychometric analysis comparing three, brief, frailty screening tools, the VIP (VIP-3 and VIP-4), CFS and PRISMA-7 in an ED setting. This study found that while all instruments had at least good accuracy for diagnosing frailty as determined by an independent multi-component CGA, the CFS and PRISMA-7 were more accurate and had greater sensitivity for frailty than either version of the VIP. Although the CFS and PRISMA-7 had generally higher predictive validity for a range of adverse health outcomes, none were statistically better and only the CFS more accurately predicted admission to hospital (i.e., ED conversion), compared with the other instruments. This overall, relatively poor, predictive ability of short risk-prediction instruments including frailty screens for mortality [38], and other adverse outcomes such as re-admission and prolonged LOS is well-established in other settings [39]. It also compares with studies in the ED, which showed that predictive validity for poor discharge outcomes including mortality were at best fair (AUC of 0.72–0.75) for measures including the CFS and Fried Frailty Phenotype [40]. In our study, predictive accuracy for readmission to hospital within 30–90 days was poor, varying between 0.60 for the PRISMA-7 and VIP to 0.69 for the CFS. A recent systematic review has likewise shown that short frailty screens have particularly poor predictive accuracy for future healthcare use (re-admission to hospital ED) [41].

The results here however, suggest that the VIP screening tool has both poor diagnostic and predictive validity. The VIP is increasingly being used to screen for frailty with some hospitals incorporating it into the triage document in the ED [42,43]. Previous studies have suggested that the VIP may not be sensitive enough for this, under-recognising frail patients with reduced mobility, acute confusion and polypharmacy, though the design of those papers did not allow them to confirm this [43]. Our results clearly show this through a robust psychometric evaluation, also supporting other studies showing that the CFS and PRISMA-7 are highly accurate in identifying frailty in ED as defined by a ‘gold standard’, CGA. This is similar to a study conducted by this research group, comparing the ISAR (Identification of Seniors At Risk) tool to the CFS and PRISMA-7, scored by nurses at ED triage, in a different cohort of older patients attending another Irish hospital in a different region of the country (urban-rural casemix in Western Ireland) [26]. That study found that the diagnostic accuracy for frailty was highest with the PRISMA-7 (AUC of 0.88), which was similar to the CFS (0.83); both were significantly more accurate than the ISAR (AUC 0.78) [26].

This study here also showed that the CFS and PRISMA-7 were significantly better at distinguishing pre-frail individuals from robust patients compared with either version of the VIP but that all tools performed similarly in their ability to differentiate frail from pre-frail individuals. However, there were no significant differences in diagnostic accuracy between the tools for distinguishing frail, pre-frail and robust participants where frailty was measured according the FRAIL scale (i.e., physical frailty) or the GFI (multi-dimensional frailty questionnaire), albeit both these scales were available as part of the broader battery of scales to aid the physician determine frailty status as part of the CGA. While no widely-accepted operational definition of frailty [4] or pre-frailty [44] exist, most of the limited number of papers that have defined a gold-standard for frailty in diagnostic accuracy studies have used a CGA [17,45]. Physical frailty, usually measured by the frailty phenotype and originally described by Fried [46], measures a different construct [47]. This likely reflects the nature of the CGA used in this study, which focused more on the multi-component deficit accumulation model of frailty and did not include a physical battery of tests such as grip strength, walking speed or gait assessment. This said, a physiotherapist assessed the patients and questions relating to physical frailty including the FRAIL scale were available in the CGA and contributed to the diagnosis. Further, concordance did indeed vary markedly between the instruments, also likely reflecting that they measure different aspects of frailty (e.g., the FRAIL scale solely measures physical frailty and the GFI measures multi-dimensional deficit aspects of frailty including cognition, function and psychosocial factors).

There are several strengths and limitations. A strength of this study was that information on outcomes such as admission rates and deaths was also complete for all screened patients and the hospital records offer reliable data. The CGA was conducted independently, supported by nutritional, cognitive and functional assessments including two frailty measures, albeit the authors acknowledge that there is as yet no widely-agreed gold standard criterion for diagnosing frailty [4,48]. Limitations include that just less than half (46%) of potential patients were available and included due to logistical issues outside of core working hours, potentially reducing the generalisability of findings. That it was conducted in a single center with a homogenous sample of community-dwelling older adults in Ireland, likely further limits this. The AD-8 questionnaire was the sole cognitive assessment used in this study, which may have limited the ability to measure cognitive frailty. This said, the AD-8 has good to excellent diagnostic accuracy for cognitive impairment across healthcare settings [49]. Another limitation is that the window for follow-up was only three months, during which time there was only 14 deaths such that a longer follow-up may have revealed significant differences in predictive accuracy between the instruments. Finally, a further evaluation of frailty was not conducted. Hence, additional study is now required to better assess the long-term predictive validity of these screens in ED.

## 5. Conclusions

This study shows that the CFS and PRISMA-7 were more accurate and should be considered in preference to using the VIP, either the 3 or 4-item version, as a brief screening instrument for frailty in ED prior to admission to hospital and to rationalise allocation of CGA. Specifically, these instruments had excellent accuracy for detecting frailty as measured by a traditional CGA, independently conducted by a consultant geriatrician-led MDT (primary outcome), which is usually regarded as a “gold standard” for diagnosing frailty [48]. However, none of the instruments had good or excellent accuracy for detecting physical frailty and in order to capture this parallel but distinct element of frailty [47], a physical assessment such as a timed-up and go test could be considered [50] as an additional frailty screen to complement the CFS and PRISMA-7. Of the screens examined in this study, the CFS was most able to predict patients likely to be admitted, although none of the instruments were strong predictors of subsequent adverse healthcare outcomes such as mortality [38] and prolonged hospitalisation [51]. This is expected given that most approaches to identifying subsequent healthcare utilisation among community-dwellers have at best, fair accuracy [39]. This study adds to the data supporting the use of the CFS as a short, reliable and valid frailty screen for use in the ED, though more data on predictive validity for frailty and long-term healthcare outcomes is now required for frailty screening in this setting.

## Figures and Tables

**Table 1:** Characteristics of patients (*n* = 197) screened for frailty comparing those classified as frail and non-frail based on the comprehensive geriatric assessment classification.

Characteristics	Total (*n* = 197)	Frail (*n* = 98)	Non-Frail (*n* = 99)	Frail vs. Non-Frail
	Median (Q3–Q1 or %)	Median (Q3–Q1 or %)	Median (Q3–Q1 or %)	*p*-Value for Difference
Age (Years)	79 (83–73)	80 (86–76)	75 (82–72)	<0.001
Sex (Female)	45%	49%	41%	0.257
BMI	25 (28–22)	24 (28–21)	26 (29–23)	0.009
MNA-SF	12 (13–10)	11 (13–9)	13 (14–12)	<0.001
AD8	1 (2–0)	1 (3–0)	0 (1–0)	<0.001
4AT	0 (1–0)	1 (2–0)	0 (0–0)	<0.001
CBS	12 (18–3)	15 (19–9)	0 (10–0)	0.001
EQ-5D (VAS)	60 (75–45)	50 (50–30)	70 (80–60)	<0.001
GSRH (V. Good/Excellent)	20%	6%	35%	<0.001
FRAIL scale	2 (3–1)	2 (3–2)	1 (2–0)	<0.001
GFI	4 (6–2)	6 (8–5)	2 (3–2)	<0.001
CFS	4 (6–3)	6 (7–5)	3 (4–2)	<0.001
PRISMA-7	4 (5–2)	5 (6–4)	2 (3–2)	<0.001
VIP 3	1 (2–0)	2 (2–1)	0 (1–0)	<0.001
VIP 4	1 (2–0)	2 (3–1)	0 (1–0)	<0.001
FRAIL (score ≥ 3)	31%	47%	14%	<0.001
GFI (score ≥ 4)	60%	96%	24%	<0.001
CFS (score ≥ 5)	45%	77%	13%	<0.001
PRISMA-7 (score ≥ 3)	67%	98%	36%	<0.001
VIP-3 (score ≥ 2)	30%	59%	2%	<0.001
VIP-4 (score ≥ 3)	14%	29%	0%	<0.001
Admitted	66%	82%	49%	<0.001
Length of stay (if admitted) in days	7 (14–3)	9 (16–5)	4 (9–2)	0.003
Died (within 3 months of presenting)	14 (7%)	12 (12%)	2 (2%)	0.005
30-day re-admission (same hospital)	14 (7%)	9 (9%)	5 (5%)	0.283
90-day re-presentation ED /re-admission *	55 (28%)	32 (33%)	23 (23%)	0.155

BMI—Body Mass Index; MNA-SF—Mini-Nutritional Assessment-short form; AD8—Alzheimer’s Disease 8 (screening test for dementia); 4AT—screening test for delirium; AMTS—Abbreviated mental Test Score; CBS—Caregiver Burden Score; EQ-5D-VAS—Euroqol EQ-5D Visual Analogue Scale; General Self-Rated Health very good or excellent—GSRH (Vg/Ex); CFS—Clinical Frailty Scale; GFI—Groningen Frailty Indicator; PRISMA-7—Programme of Research to Integrate Services for the Maintenance of Autonomy 7; VIP—Variable Indicative of Placement risk. * To local model 3 or 4 hospital

**Table 2:** Agreement between frailty scales, frailty cut-offs and comprehensive geriatric assessment frailty status according to Spearman’s correlation (r_s_) for the number of criteria, and Cohen’s kappa (κ) for established, binary frailty cut-off values.

**Agreement (r_s_) between Frailty Scales (Number of Criteria)**						
**Frailty Scales (cut-offs)**	**CFS** **(Scale 1–9)**	**PRISMA-7** **(Scale 0–7)**	**VIP-3** **(Scale 0–3)**	**VIP-4** **(Scale 0–4)**	**FRAIL** **(Scale 0–5)**	**GFI** **(Scale 0–15)**
CFS (scale 1–9)	-	0.70 **	0.56 **	0.58 **	0.52 **	0.62 **
PRISMA-7 (scale 0–7)	0.70 **	-	0.48 **	0.51 **	0.53 **	0.63 **
VIP-3 (scale 0–3)	0.56 **	0.48 **	-	0.98 **	0.44 *	0.59 **
FRAIL (scale 0–5)	0.52 **	0.53 **	0.44 *	0.47 *	-	0.59 **
GFI (scale 0–15)	0.62 **	0.63 **	0.59 **	0.61 **	0.59 **	-
**Agreement (κ) between Frailty Cut-Offs and Comprehensive Geriatric Assessment**						
**Frailty Scales (cut-offs)**	**CFS** **(Frail ≥ 5)**	**PRISMA-7** **(Frail ≥ 3)**	**VIP-3** **(Frail ≥ 2)**	**VIP-4** **(Frail ≥ 3)**	**FRAIL** **(Frail ≥ 3)**	**GFI** **(Frail ≥ 4)**
CFS (frail ≥ 5)	-	0.48 **	0.46 **	0.25 **	0.26 **	0.50 **
PRISMA-7 (frail ≥ 3)	0.48 **	-	0.29 **	0.15 **	0.26 **	0.59 **
VIP-3 (frail ≥ 2)	0.46 **	0.29 **	-	0.56 **	0.16 *	0.41 **
FRAIL (frail ≥ 3)	0.26 **	0.26 **	0.16 *	0.21 *	-	0.29 **
GFI (frail ≥ 4)	0.50 **	0.59 **	0.41 **	0.17 **	0.29 **	-
CGA (frail/non-frail)	0.64 **	0.62 **	0.56 **	0.29 **	0.32 **	0.72 **

CFS—Clinical Frailty Scale; CGA—Comprehensive Geriatric Assessment; GFI—Groningen Frailty Indicator; PRISMA-7—Programme of Research to Integrate Services for the Maintenance of Autonomy 7; VIP—Variable Indicative of Placement risk, 3-item (VIP-3) or 4-item (VIP-4). Note: * *p* < 0.05, ** *p* ≤ 0.001

**Table 3:** Sensitivity, specificity, positive predictive value (PPV), negative predictive value (NPV), positive (PLR) and negative likelihood ratio (NLR) with 95% confidence intervals (CI), for the Clinical Frailty Scale (CFS), PRISMA-7 and the three and four-item versions of the Variable Indicative of Placement risk (VIP) in their ability to differentiate frail from non-frail based on an independent comprehensive geriatric assessment.

Frailty Screen Cut-Off	Youden’s Index	Sensitivity (95% CI)	Specificity (95% CI)	PPV (95% CI)	NPV (95% CI)	False Positive (95% CI)	False Negative (95% CI)	PLR † (95% CI)	NLR † (95% CI)
**CFS**									
≥2	0.15	1 (0.92–1.0)	0.15 (0.09–0.24)	0.53 (0.46–0.61)	1 (0.75–1.0)	0.47 (0.39–0.54)	0 (0–0.25)	1.1 (0.93–1.4)	0 (0.0–0.0)
≥3	0.39	1 (0.95–1.0)	0.39 (0.30–0.49)	0.61 (0.53–0.69)	1 (0.89–1.0)	0.39 (0.31–0.47)	0 (0–0.11)	1.6 (1.3–2.0)	0 (0–0)
≥4 ^	0.66	0.95 (0.88–0.98)	0.70 (0.60–0.79)	0.76 (0.67–0.83)	0.93 (0.84–0.98)	0.25 (0.17–0.33)	0.07 (0.02–0.16)	3.1 (2.2–4.3)	0.07 (0.03–0.17)
≥5 *	0.64	0.77 (0.67–0.85)	0.87 (0.78–0.93)	0.85 (0.76–0.92)	0.80 (0.71–0.87)	0.15 (0.08–0.24)	0.20 (0.13–0.29)	5.8 (3.5–9.6)	0.25 (0.17–0.37)
≥6	0.50	0.53 (0.42–0.63)	0.97 (0.91–0.99)	0.94 (0.84–0.99)	0.68 (0.59–0.75)	0.06 (0.01–0.16)	0.32 (0.25–0.41)	17 (5.6–51)	0.47 (0.37–0.61)
≥7	0.25	0.27 (0.19–0.37)	0.98 (0.92–1.0)	0.93 (0.75–0.99)	0.58 (0.50–0.65)	0.07 (0.01–0.25)	0.42 (0.35–0.50)	13 (3.4–49.6)	0.72 (0.6–0.87)
≥8	0.01	0.03 (0.01–0.09)	0.98 (0.92–1.0)	0.60 (0.17–0.93)	0.51 (0.44–0.58)	0.4 (0.07–0.83)	0.49 (0.42–0.56)	1.5 (0.41–5.5)	0.96 (0.82–1.12)
**PRISMA-7**									
≥2	0.22	1 (0.95–1.0)	0.23 (0.15–0.33)	0.56 (0.48–0.63)	1 (0.82–1.0)	0.44 (0.37–0.52)	0 (0.0–0.18)	1.3 (1.0–1.6)	0 (0–0)
≥3*	0.62	0.98 (0.92–0.99)	0.64 (0.54–0.73)	0.73 (0.64–0.80)	0.97 (0.86–0.99)	0.27 (0.20–0.36)	0.03 (0.01–0.11)	2.7 (2–3.6)	0.03 (0.01–0.12)
≥4 ^	0.68	0.87 (0.78–0.92)	0.82 (0.73–0.89)	0.83 (0.74–0.89)	0.86 (0.77–0.92)	0.17 (0.11–0.26)	0.14 (0.08–0.23)	4.7 (3.1–7.3)	0.16 (0.1–0.26)
≥5	0.56	0.64 (0.54–0.74)	0.92 (0.84–0.96)	0.89 (0.78–0.95)	0.72 (0.64–0.80)	0.11 (0.05–0.22)	0.28 (0.20–0.36)	7.9 (4.1–15.2)	0.38 (0.29–0.51)
≥6	0.28	0.31 (0.22–0.41)	0.97 (0.91–0.99)	0.91 (0.75–0.98)	0.59 (0.51–0.66)	0.09 (0.02–0.25)	0.41 (0.34–0.49)	10 (3.4–29.6)	0.7 (0.6–0.85)
≥7	0.04	0.04 (0.01–0.11)	1 (0.95–1.0)	1 (0.40–1.0)	0.52 (0.44–0.59)	0 (0–0.60)	0.49 (0.41–0.56)	NA	0.94 (0.8–1.1)
**VIP-3**									
≥1	0.43	0.87 (0.78–0.92)	0.57 (0.46–0.66)	0.66 (0.57–0.74)	0.81 (0.70–0.89)	0.34 (0.26–0.43)	0.19 (0.11–0.30)	2.0 (1.5–2.6)	0.23 (0.14–0.38)
≥2 * ^	0.57	0.58 (0.48–0.68)	0.98 (0.92–0.99)	0.97 (0.87–0.99)	0.70 (0.62–0.78)	0.03 (0.01–0.13)	0.29 (0.22–0.38)	28.5 (7.3–111)	0.42 (0.33–0.55)
≥3	0.15	0.14 (0.08–0.23)	1 (0.95–1.0)	1 (0.73–1.0)	0.54 (0.47–0.61)	0 (0–0.27)	0.46 (0.39–0.53)	NA	0.85 (0.72–1.0)
**VIP-4**									
≥1	0.42	0.87 (0.78–0.92)	0.56 (0.45–0.65)	0.66 (0.57–0.74)	0.81 (0.69–0.89)	0.34 (0.26–0.43)	0.19 (0.11–0.31)	1.9 (1.5–2.5)	0.24 (0.14–0.39)
≥2 ^	0.61	0.63 (0.53–0.73)	0.98 (0.92–0.99)	0.97 (0.88–0.99)	0.73 (0.64–0.80)	0.03 (0.01–0.12)	0.27 (0.20–0.36)	31 (8–121)	0.37 (0.28–0.49)
≥3 *	0.29	0.29 (0.20–0.39)	1 (0.95–1.0)	1 (0.85–1.0)	0.59 (0.51–0.66)	0 (0–0.15)	0.41 (0.34–0.49)	NA	0.71 (0.59–0.85)
≥4	0.05	0.05 (0.02–0.12)	1 (0.95–1.0)	1 (0.46–1.0)	0.52 (0.44–0.59)	0 (0–0.54)	0.48 (0.41–0.55)	NA	0.94 (0.8–1.1)

* Recommended cut-off score for frailty; ^ Optimal cut-off based on Youden’s Index; † Prevalence adjusted; NA = Not available; Note: PRISMA-7 & CFS start at ≥ 2 since no participants scored 1 or less.

**Table 4:** Diagnostic and predictive accuracy for different outcomes based on area under the curve (AUC) scores for the Clinical Frailty Scale (CFS), the Programme of Research to Integrate Services for the Maintenance of Autonomy 7 (PRISMA-7) tool, the Variable Indicative of Placement risk, three item (VIP-3) and four-item (VIP-4) versions.

Diagnostic Approach	CFS	PRISMA-7	VIP-3	VIP-4
AUC (95% confidence intervals) for differentiating frail from non-frail				
CGA	0.91 (0.87–0.95) *p* < 0.001	0.90 (0.86–0.95) *p* < 0.001	0.84 (0.78–0.89) *p* < 0.001	0.84 (0.79–0.90) *p* < 0.001
FRAIL scale i.e., physical frailty (frail ≥ 3 criteria)	0.69 (0.62–0.77) *p* < 0.001	0.70 (0.62–0.77) *p* < 0.001	0.63 (0.55–0.71) *p* = 0.004	0.66 (0.57–0.74) *p* < 0.001
GFI (frail ≥ 4 criteria)	0.82 (0.76–0.88) *p* < 0.001	0.85 (0.79–0.91) *p* < 0.001	0.78 (0.71–0.84) *p* < 0.001	0.78 (0.72–0.85) *p* < 0.001
AUC (95% confidence intervals) for differentiating frail from pre-frail				
CGA	0.83 (0.75–0.91) *p* < 0.001	0.83 (0.75–0.91) *p* < 0.001	0.82 (0.75–0.89) *p* < 0.001	0.83 (0.77–0.90) *p* < 0.001
FRAIL scale (physical frailty)	0.62 (0.53–0.71) *p* = 0.012	0.61 (0.52–0.70) *p* = 0.021	0.55(0.46–0.65) *p* = 0.275	0.59 (0.49–0.68) *p* = 0.077
CGA (pre-frail/robust)	0.81 (0.72–0.89) *p* < 0.001	0.82 (0.73–0.90) *p* < 0.001	0.57 (0.45–0.68) *p* = 0.260	0.56 (0.44–0.67) *p* = 0.325
FRAIL scale (i.e., physical frailty)	0.78 (0.69–0.86) *p* < 0.001	0.78 (0.70–0.86) *p* < 0.001	0.74 (0.66–0.82) *p* < 0.001	0.75 (0.67–0.83) *p* < 0.001
AUC (95% confidence intervals) for predicting adverse health-related outcomes				
Outcomes (Yes/No)	CFS	PRISMA-7	VIP-3	VIP-4
Admission	0.78 (0.70–0.85) *p* < 0.001	0.65 (0.56–0.74) *p* < 0.001	0.59 (0.51–0.67) *p* = 0.032	0.60 (0.52–0.68) *p* = 0.026
Length of Stay (>7 days, i.e., median LOS)	0.60 (0.51–0.70) *p* = 0.044	0.61 (0.51–0.70) *p* = 0.036	0.66 (0.57–0.76) *p* = 0.001	0.68 (0.58–0.77) *p* < 0.001
Readmission to hospital (within 30–90 days)	0.69 (0.61–0.78) *p* = 0.005	0.60 (0.49–0.72) *p* = 0.130	0.60 (0.47–0.73) *p* = 0.149	0.60 (0.47–0.74) *p* = 0.125
Died within 3 months of presentation	0.71 (0.59–0.83) *p* = 0.008	0.71 (0.58–0.84) *p* = 0.009	0.66 (0.50–0.82) *p* = 0.050	0.67 (0.51–0.83) *p* = 0.036

CFS—Clinical Frailty Scale; CGA—Comprehensive Geriatric Assessment; GFI—Groningen Frailty Indicator; PRISMA-7—Programme of Research to Integrate Services for the Maintenance of Autonomy 7; VIP—Variable Indicative of Placement risk, 3-item (VIP-3) or 4-item (VIP-4).

## Data Availability

Data are available on request.
